# Hypersensitivity Pneumonitis: Diagnostic and Therapeutic Challenges

**DOI:** 10.3389/fmed.2021.718299

**Published:** 2021-09-23

**Authors:** Maria Laura Alberti, Emily Rincon-Alvarez, Ivette Buendia-Roldan, Moises Selman

**Affiliations:** ^1^Hospital María Ferrer, Buenos Aires, Argentina; ^2^Fundación Neumológica Colombiana, Bogotá, Colombia; ^3^Instituto Nacional de Enfermedades Respiratorias “Ismael Cosío Villegas”, Mexico City, Mexico

**Keywords:** lung fibrosis, risk factors, hypersensitivity pneumonitis, prognostic factors, diagnosis

## Abstract

Hypersensitivity pneumonitis (HP) is one of the most common interstitial lung diseases (ILD), that presents unique challenges for a confident diagnosis and limited therapeutic options. The disease is triggered by exposure to a wide variety of inciting antigens in susceptible individuals which results in T-cell hyperactivation and bronchioloalveolar inflammation. However, the genetic risk and the pathogenic mechanisms remain incompletely elucidated. Revised diagnostic criteria have recently been proposed, recommending to classify the disease in fibrotic and non-fibrotic HP which has strong therapeutic and outcome consequences. Confident diagnosis depends on the presence of clinical features of ILD, identification of the antigen(s), typical images on high-resolution computed tomography (HRCT), characteristic histopathological features, and lymphocytosis in the bronchoalveolar lavage. However, identifying the source of antigen is usually challenging, and HRCT and histopathology are often heterogeneous and not typical, supporting the notion that diagnosis should include a multidisciplinary assessment. Antigen removal and treating the inflammatory process is crucial in the progression of the disease since chronic persistent inflammation seems to be one of the mechanisms leading to lung fibrotic remodeling. Fibrotic HP has a few therapeutic options but evidence of efficacy is still scanty. Deciphering the molecular pathobiology of HP will contribute to open new therapeutic avenues and will provide vital insights in the search for novel diagnostic and prognostic biomarkers.

## Introduction

Hypersensitivity Pneumonitis (HP) is an immune-mediated disease that manifests as interstitial lung disease (ILD) in susceptible individuals after exposure to identified or unidentified inciting agent(s) (1). The disease has a heterogeneous clinical presentation, as well as varied radiological and morphological patterns likely associated with the individual genetic susceptibility, type of antigen, the extent of exposure, and the interaction with other injuring factors (2, 3).

The genetic susceptibility that increases the risk to develop the disease is unclear, and most studies have focuses on polymorphisms in the Major Histocompatibility Complex class II (HLA-DR and HLA-DQ) molecules which are involved in the presentation of antigens by antigen-presenting cells (APCs) and recognized by the respective T-cell receptor on the CD4+ T-cell surface (2–5). More recently, it was found that several interactions involving polymorphisms of either the *SFTPA1* and/or *SFTPA2*, increase HP risk whereas their interactions with the hydrophobic surfactant proteins (*SFTPB* and *SFTPC*) were associated with a decreased risk to develop the disease (6).

On the other hand, exposure to damaging agents, such as cigarette smoke, air pollution, viral infections, and pesticides also influences the development of the disease as well as the heterogeneous behavior (2, 3, 7–9).

## Epidemiology

The definite incidence and prevalence of HP are uncertain because it varies according to the countries and customs, and importantly due to the lack of consensus over a definition of the disease, and the inability to detect the source of antigen exposure leading to a misdiagnosis attributing the patient's findings to another ILD. The incidence of HP has been reported in some countries such as the UK population, where is recorded as ~1 per 100,000 (10). In the US, a yearly incidence in the range of 1.7–2.7 per 100,000 population, has been recently reported (11), while Japan and France estimate an incidence between 0.3 and 0.9 per 100,000 individuals (12, 13). However, the incidence could be much higher according to one study that reported bird breeder's disease in 4.9 per 100,000 individuals over 10 years or 54.6 per 100,000 bird breeders (1, 14). The proportion of HP among all ILD cases could be higher and may represents around half of the newly diagnosed patients in high prevalent regions. The high variability in incidence and prevalence likely depends of many factors including differences in geographical conditions, local customs and occupational factors of each region and also because only until recently, there is a consensus over a definition of the disease.

## Antigens and Sources of Exposure

Numerous antigens able to induce the disease have been identified and the list is constantly being expanded. The most studied antigens are avian antigens, fungi and thermophilic bacteria in the home or the working environment (15, 16); but there are also numerous reports revealing the association with other type of bacteria, protozoal, other animal proteins, and low-molecular-weight chemical compounds. For this reason, it is very important to investigate the presence of visible mold indoors; occupational environments such as where greenhouses, mushroom farming, compost, other food production methods, and metalworking fluids that could be contaminated by bacterial, mycobacterial, and fungal organisms (17). Even hobby activities may be a source of HP antigens, for example, non-tuberculous mycobacteria have been identified in patients exposed to indoor hot tubs and outdoor pools (18). Finally, specific chemicals used in industry, such as isocyanates and anhydrides, should also be considered as causal antigens. A list with most of the antigens and sources of exposure identified so far can be found in ATS/JRS/ALAT Guidelines (1).

## Pathogenic Mechanisms

### The Inflammatory Response

The first step is the sensitization to the inhaled antigens which is associated with repeated exposure in individuals with genetic susceptibility to HP. The immunopathological response to the antigens involves T- and B-cells. Progression from sensitization to HP requires the accumulation of CD4+ TH1 cells in the lung, creating a pro-inflammatory microenvironment. Importantly, the suppressive activity of regulatory T cells is impaired, resulting in the amplification of the inflammatory response. IFNγ and TNF promote the accumulation, activation, and aggregation of macrophages, resulting in the development of granulomatous inflammation (4, 19). Also, immune complex-mediated lung injury with specific IgG antibodies may contribute to the inflammatory response.

### The Fibrotic Response

Several factors may hamper the resolution of the inflammation, including further exposure to the antigen, which occurs mainly when it has not been identified (20), cigarette smoking, a genetic predisposition that may enhance the development of autoantibodies (21), and other unknown factors.

Several changes in T cell subsets are found in fibrotic HP, which may contribute to the non-resolution of inflammation triggering a fibrotic response, including a decrease of the immunoregulatory and antifibrotic γδ T cells, an increase of CD4+ cells, and a switch from a predominant TH1-like phenotype to a TH2-like phenotype (4, 19). TH2 cells secrete, among others, IL-4 and mainly IL-13 that contribute to a fibrotic response stimulating the TGFβ1 signaling pathway and activating the expansion of fibroblasts population (19, 22, 23). Fibroblasts arrive at the injured areas and differentiate into myofibroblasts, which are responsible for the accumulation of extracellular matrix. At the initial stages of fibrosis, the disease may stabilize or even improve in the pulmonary functional status, however, a subset of patients develops an aggressive phenotype called progressive pulmonary fibrosis that results in the destruction of the lung architecture (24). The mechanisms triggering this devastating phenotype are unclear but may include the type of fibrosis (UIP vs. non-UIP pattern), the aberrant composition and stiffness of the extracellular matrix, and the emergence of some unique profibrotic cell subsets (25).

## Clinical Features

It has been recently proposed that HP can be classified in fibrotic and non-fibrotic phenotypes (1). This proposal was considered to be more consistently associated with the clinical course, outcomes, and treatment efficiency.

Dyspnea is the main symptom of both non-fibrotic and fibrotic HP. Occasionally, patients with the non-fibrotic disease may present an acute influenza-like syndrome occurring a few hours after a (usually) substantial exposure. In these cases, symptoms gradually decrease over hours/days but may recur with re-exposure. More often, patients with non-fibrotic HP present progressive dyspnea during weeks or a few months together with constitutional symptoms, including fever, chills, chest tightness, wheezing, and weight loss (3). Patients with fibrotic HP show progressive (usually insidious) exertional dyspnea and chronic cough that develops over months to years. Clubbing may be present and on auscultation may yield inspiratory “velcro” crackles. Some patients display a high-pitched wheeze at the end of inspiration (“chirping” rales) while others describe the presence of inspiratory squeaks, caused by airways involvement (26). Pulmonary function test (PFT) reveal in both fibrotic and non-fibrotic HP a predominantly restrictive defect with DL_CO_ impairment, although some small airway obstruction may be detected in non-fibrotic patients. Finally, in the advanced stage of the disease patients may develop pulmonary arterial hypertension which is more prevalent in hypoxemic patients with greater impairment in lung function and lower exercise capacity (27).

## Diagnostic Approach

HP represents a diagnostic challenge and requires a high index of suspicion by the clinician evaluating by the first time a patient with ILD (28, 29). Targeted diagnostic steps should include a thorough evaluation of the ILD patient's history of occupational and environmental antigenic exposures, chest high resolution computed tomography (HRCT), serum specific IgGs for confirmation of exposure or as a screening tool, bronchioalveolar lavage (BAL), and histopathological study in some cases (1, 30, 31).

### Evidence of Exposure

Identification of the source of exposure and putative antigen(s) can be difficult. Validated and regionally relevant questionnaires that include occupational, residential, and avocational environments are mandatory (30, 32). Questions should also consider indirect exposures through contact with individuals who may carry antigens on their clothing or other materials. If an exposure is identified, details of duration, extent, and frequency should be obtained and importantly putative cause-effect relationship with symptoms. Evidence-based guidance has been published by WHO suggesting questions that may help clinicians to find out indoor dampness and molds (33). The on-site visual inspection is also useful for identifying obvious exposure sources (30).

### Diagnostic Detection of Cellular and Humoral Immune Responses to HP Antigens

Identification of serum-specific Immunoglobulins (ssIGg) may help to recognize the inciting antigen (1, 30). According to the ATS Workshop Report, serum IgG testing against potential antigens associated with HP distinguish this disease from other ILDs with a sensitivity and specificity of 83 and 68%, respectively (30). However, it is important to emphasize that the presence of positive circulating antibodies, is only evidence of exposure to a potential HP antigen but does not prove causality and it may be worthy of further consideration to explore the source (30, 34).

On the other hand, since antigen T-cell mediated immune response plays a pivotal role in the pathogenesis of HP it has been proposed that lymphocyte proliferation testing may be a diagnostic tool (30, 35, 36).

However, studies using this method are scant, usually performed in small cohorts, and primarily in patients suspected to have bird-related HP. In addition, there is no standardized methodology to recommend in clinical practice. Nevertheless, this technique may be a promissory diagnostic tool in the future, mainly in patients with fibrotic HP that do not have detectable antibodies to causative antigens (37).

### Chest HRCT Scanning

HRCT plays a pivotal role in the diagnosis of HP. In both fibrotic and non-fibrotic HP, images should be acquired at deep inspiration and after prolonged expiration.

**Figure 1:**
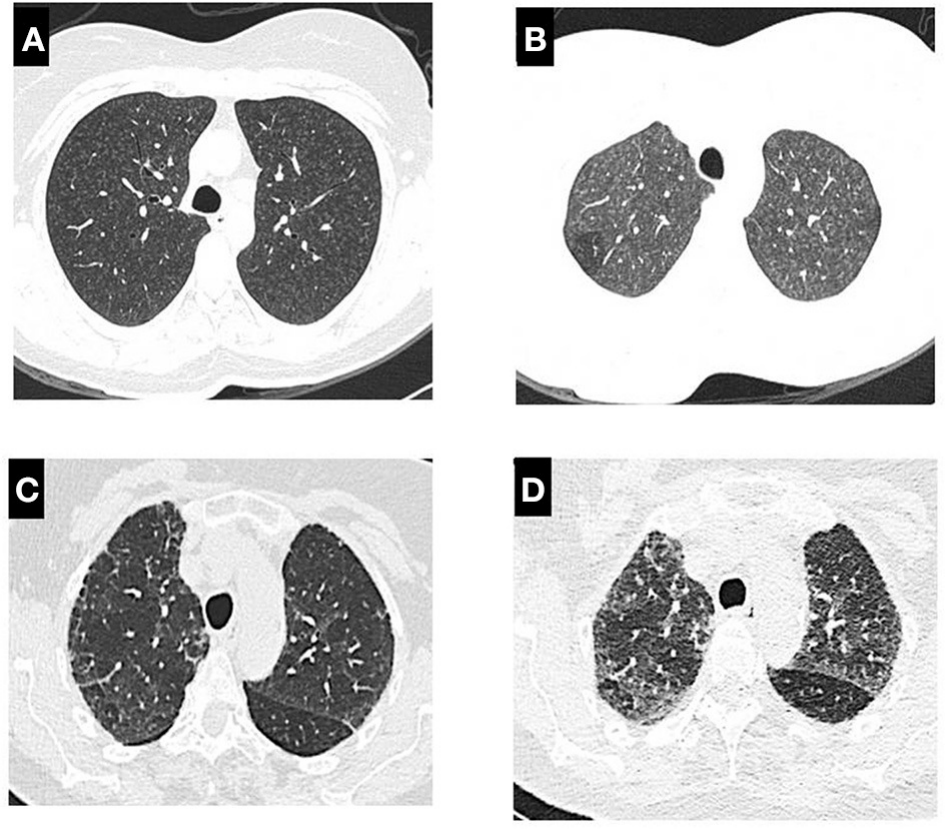
High-resolution computed tomography scan in patients with non-fibrotic HP: **(A)** show inspiratory phase with diffuse centrilobular nodules, **(B)** expiratory phase in the same patient with centrilobular nodules and air trapping in right side; **(C)** in inspiratory phase present subpleural reticular pattern, with mosaic attenuation which is highlighted in the expiratory phase **(D)**.

**Figure 2:**
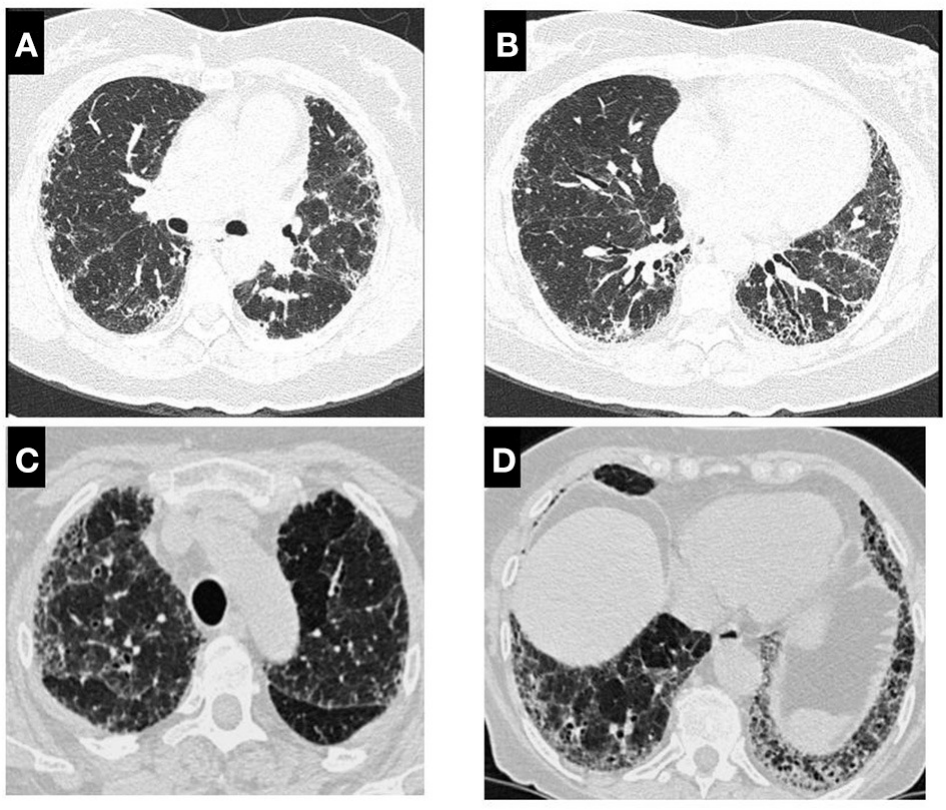
High-resolution computed tomography scan in patients with fibrotic HP. In **(A)**, it is observed bilateral subpleural reticulation and in **(B)** traction bronchiectasis, honey combing and discrete ground-glass opacification and volume loss in left lung; **(C)** show lung fibrosis and areas of low attenuation and the same patient in **(D)** bronchiectasis and persistence of mosaic attenuation.

The presence of centrilobular nodules, ground-glass opacities, mosaic attenuation, air trapping, mosaic perfusion are recognized as the principal findings in both fibrotic a non-fibrotic HP (Figure 1). The “three-density pattern” which describes a form of mosaic attenuation that combines areas of ground-glass opacification, lobular areas of low attenuation, and normal lung has a specificity of 93% for a diagnosis of fibrotic HP (38). For the fibrotic HP pattern, coexisting lung fibrosis and inflammation with signs of bronchiolar obstruction are highly suggestive. Honeycombing and traction bronchiectasis can be present and may be extensive in severe forms of fibrotic HP. Lung fibrosis can be more severe in the mid or mid and lower lung zones or equally distributed in the three lung zones with relative basal sparing (Figure 2) (39).

In general terms, the ATS/JRS/ALAT Guidelines and CHEST Guideline and Expert Panel Report 2021, show similar recommendations to classify HRCT images in the context of fibrotic or non-fibrotic HP (1, 31).

### Cell Profile in the Bronchoalveolar Lavage Fluid (BAL)

**Figure 3:**
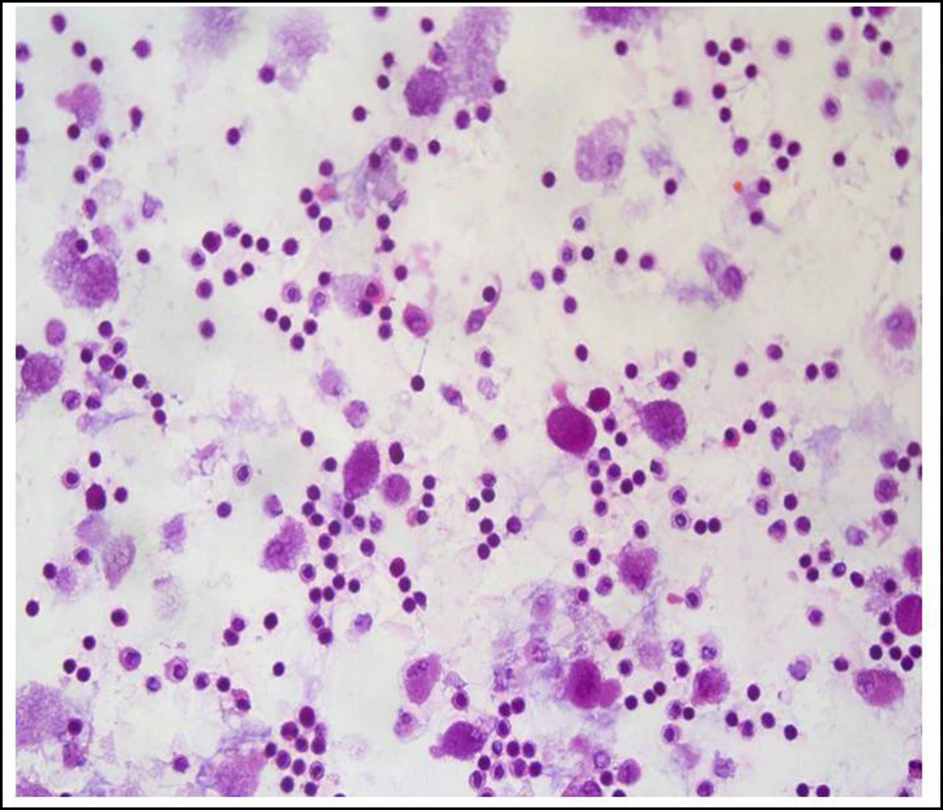
Differential cell count in bronchoalveolar lavage fluid from a patient with hypersensitivity pneumonitis showing strong lymphocytosis. Hematoxylin and eosin staining, magnification: 40×.

BAL is a safe and well-tolerated diagnostic tool to evaluate alveolar inflammation. Increased cellularity with lymphocytosis is an important piece to improve the diagnostic likelihood of HP, where a higher percentage of lymphocytes could reflect the degree of alveolitis (Figure 3). However, the threshold proportion of BAL fluid lymphocytes that distinguishes HP from non-HP ILD is unclear and is strongly associated with the presence and extent of fibrotic changes (1, 31). Important for interpretation, BAL lymphocytosis may also be influenced by several variables, including, timing relative to antigen exposure, smoking status, and others (40). In general, and according to the ATS/JRS/ALA and CHEST guidelines, and to our own experience, we consider that a 30% threshold is reasonable for use in the differential diagnosis of HP vs. non-HP ILD.

### Lung Biopsy

When the diagnosis is uncertain even after multidisciplinary discussion, lung biopsy is indicated. The histological specimen can be obtained by transbronchial cryobiopsy (if the Institution has experience with this technique), or surgical lung biopsy where samples of two different lobes are indicated (41–43).

**Figure 4:**
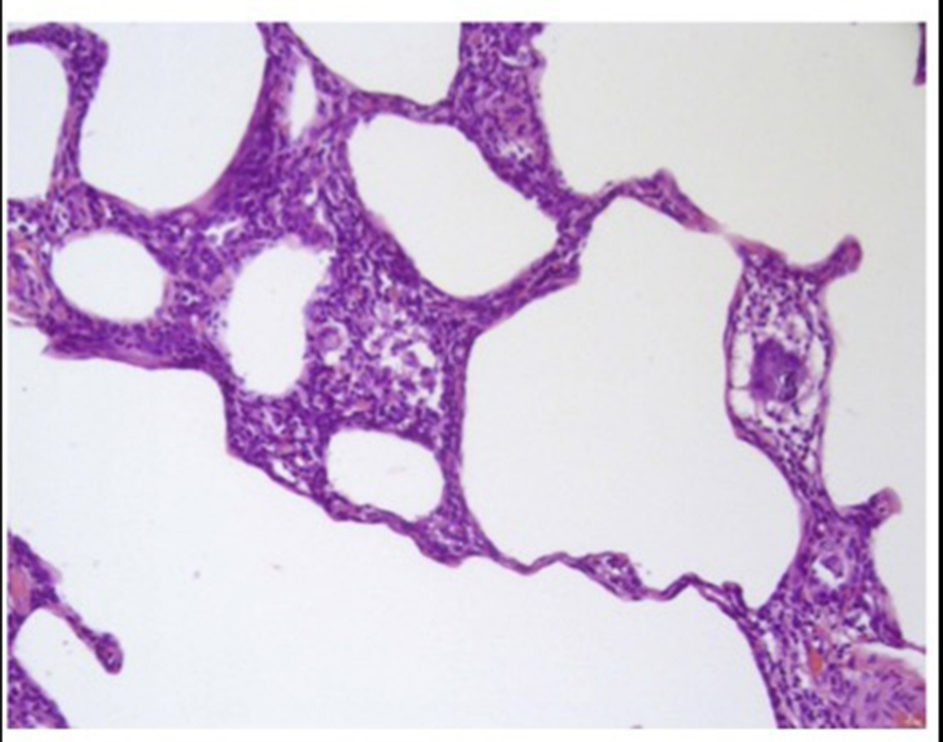
Lung biopsy sample from a patient with non-fibrotic hypersensitivity pneumonitis showing cellular chronic interstitial pneumonia and a poorly formed non-necrotizing granuloma. Hematoxylin and eosin staining, magnification: 40×.

The histopathological features vary according to the phenotypes. In the case of the non-fibrotic HP, characteristic findings include bronchiolocentric cellular interstitial pneumonia and cellular bronchiolitis of lymphocyte-predominant inflammatory infiltrate, as well as loosely formed granulomas and randomly scattered multinucleated giant cells within the interstitial inflammation (Figure 4) (1, 31).

Fibrotic HP differs from non-fibrotic HP in that the underlying chronic interstitial pneumonia and/or bronchiolitis is complicated by fibrosis, which occasionally may overlap with a UIP pattern hindering the differential diagnosis with IPF. In other cases, interstitial pneumonia shows a more uniform and diffuse distribution mimicking fibrotic non-specific interstitial pneumonia or may display features compatible with interstitial airway-centered fibrosis (1, 31, 44). Findings of non-fibrotic HP may help to distinguish fibrotic HP from other fibrotic lung disorders. Moreover, UIP-like pattern is related with worst survival (1, 45, 46).

### Multidisciplinary Discussion

**Figure 5:**
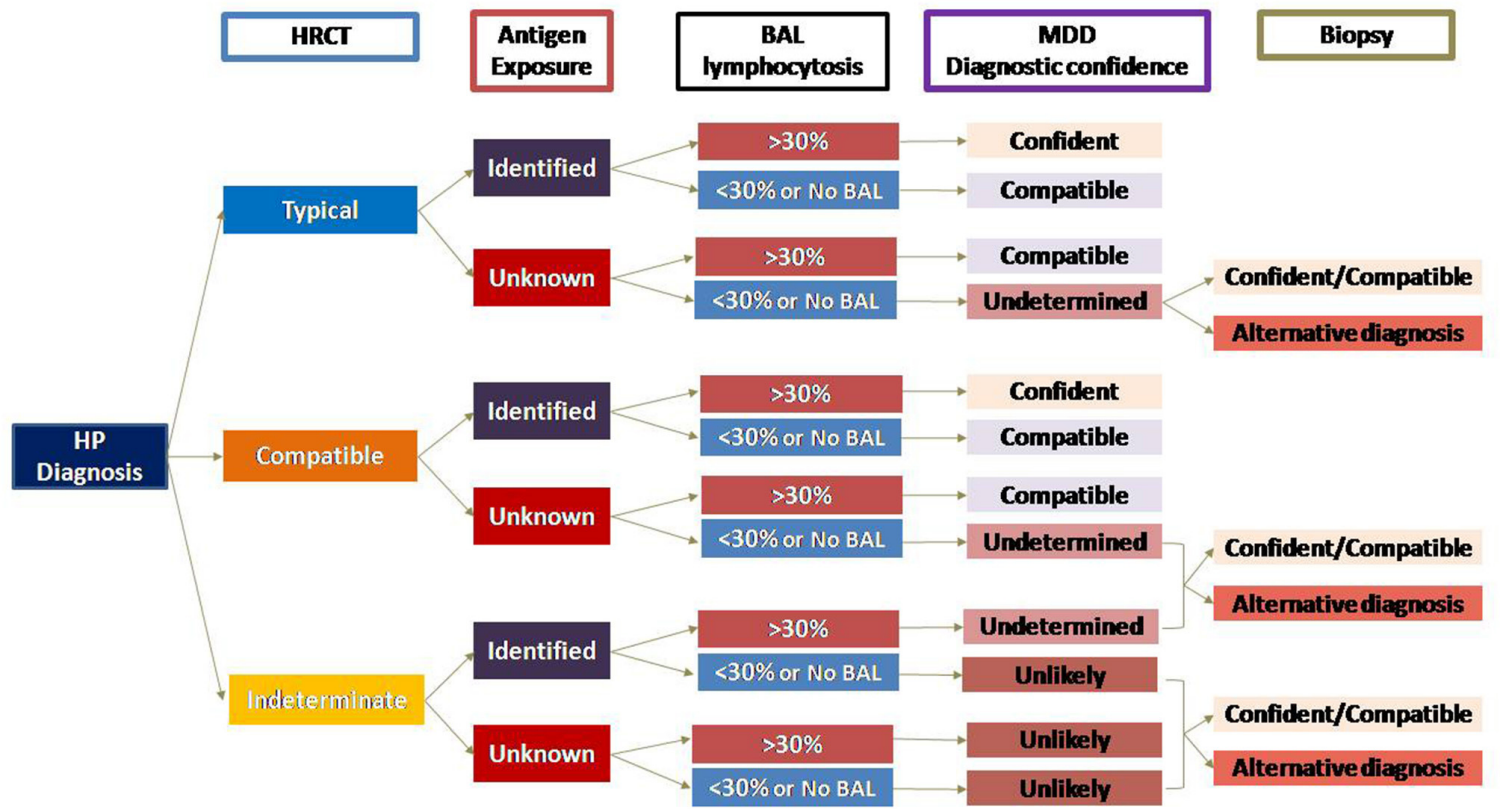
Hypersensitivity pneumonitis diagnostic algorithm. The diagnosis of HP diagnosis relies primarily on three domains: HRCT pattern (according to ATS guideline classification), antigen exposure, and BAL lymphocytosis. This approach is followed by multidisciplinary team discussion where the diagnostic confidence is made. Undetermined or Unlikely HP may require the lung biopsy to orientate to an alternative diagnosis, or occasionally, reveal a hidden HP. Diagnostic confidence: Confident (>90%), Compatible (70%-89%), Undetermined (50%-69%), Unlikely (<50%). NO BAL (Not performed, e.g., patients with comorbidities and/or very low pulmonary function tests, patient's refusal to do the procedure; BAL not available, or other reasons). HP, hypersensitivity pneumonitis; HRCT, high resolution computed tomography; BAL, bronchoalveolar lavage; MDD, multidisciplinary discussion.

As recommended in all newly diagnosed ILD, multidisciplinary evaluation of patients with suspected HP is advised. Diagnosis is guided by the integration of clinical history and questionnaire, environmental assessment and sampling, HRCT, and BAL, and in select cases, immunologic testing, and histopathological evaluation, which likely will provide the most precise approach to diagnosis. Two recently published guidelines, from ATS/JRS/ALAT (1), and from CHEST (31) recommended diagnostic algorithms based in three domains: exposure identification, HRCT findings, and BAL lymphocytosis, which in the case of the ATS/JRS/ALAT diagnostic criteria is strengthened by histopathologic findings. A recent study showed that the agreement between them in a real-life setting was low for definitive/high-confidence diagnosis (47). Accordingly, we proposed an algorithm for the diagnostic evaluation of HP, based in the same domains used by both guidelines (Figure 5).

## Prognostic Factors

### HRCT and Morphological Phenotypes

The type of fibrotic structural remodeling may indicate an increased risk of early mortality. Particularly, the usual interstitial pneumonia (UIP) pattern either by HRCT or biopsy carry-out the worst prognosis. For example, a study that involved a large cohort of patients, showed that CT honeycombing is highly prevalent in diverse forms of ILD, including HP, and that is associated with marked increased in long-term mortality rate compared with those without honeycombing (48). Likewise, in another study where three radiologically defined phenotypes were identified, it was found that patients with typical UIP-like pattern (that included honeycombing) displayed a median survival similar to IPF (the most aggressive ILD), and significantly lower than in patients with non-honeycomb fibrosis (2.8 vs. 7.95 years) (49). Therefore, CT honeycombing is prevalent in fibrotic HP and identifies a progressive fibrotic phenotype that is associated with increased mortality rates.

UIP findings in the lung biopsy also predict prognosis (1, 45). Interestingly, a biomarker that distinguishes UIP from a non-UIP pattern has been proposed for the diagnosis of IPF. The Envisia Genomic Classifier, through the detection of a 190-gene machine-learning classifier in lung samples obtained from transbronchial biopsies, could assist in the confident diagnosis of UIP (50). However, if this molecular biomarker will be useful (and accessible) in UIP of other fibrotic lung disorders, such as HP, is largely unknown.

Interestingly, some HP patients may present features of pleuroparenchymal fibroelastosis (PPFE) an unusual biopathological process characterized by upper-lobe-dominant progressive pulmonary fibrosis consisting of visceral pleural thickening with collagenous matrix and subpleural elastosis (51), and this association is linked to worsened HP survival (52).

Importantly, using automated computer-based quantitative imaging it was shown that patients with a pulmonary vessel volume above 6 · 5% of the total lung volume, had a rate of disease progression, nearly identical to that of IPF (53).

### Genomic and Molecular Risk Factors

There are significant inter-individual differences in the severity and progression of the pulmonary disease in patients with HP that otherwise seem to share similar antigen exposure and other demographic characteristics indicating that some genetic or molecular modifiers may contribute.

For example, it was found that an exaggerated shortening of telomeres was associated with fibrosis and was a strong predictor of poor survival in HP patients. Moreover, short telomere length was also linked to radiographic and histopathologic changes similar to IPF (54).

More recently, it was demonstrated that around 10% of patients with HP carry rare protein-altering variants in telomere-related genes, such as *TERT, RTEL1*, and *PARN* (55). Importantly, this finding was associated with shorter peripheral blood telomere length and significantly reduced transplant-free survival.

Likewise, The MUC5B promoter polymorphism rs35705950 minor allele was associated with HRCT evidence of fibrosis and traction bronchiectasis, and in contrast to IPF, showed a statistical tendency toward poorer survival among patients with HP (54).

There is some evidence that some biomarkers may be an independent predictor of disease progression and mortality in HP. The relative change in the serum levels of KL-6/MUC1, a human mucin protein expressed by type 2 alveolar epithelial cells, is associated with rapid progression in patients with fibrotic HP (56). Interestingly, raised KL-6 is associated with early-stage HP suggesting a mechanistic link with the behavior of the lung epithelium (57).

YKL-40 is a chitinase-like protein mainly secreted by inflammatory and epithelial cells, which is involved in the inflammatory response to tissue damage. HP patients who experienced disease progression had higher baseline serum YKL-40 levels than those who remained stable during follow-up. Likewise, HP patients who died had higher baseline serum YKL-40 levels than those who survived (58).

A serum chemokine profile showed that a lower CXCL9, in combination with higher CCL17, was an important predictor of worsening lung function (59). However, is important to emphasize that studies of prognostic biomarkers in HP are scanty, performed in small cohorts, and usually without verification cohorts.

Two recent studies have demonstrated that a subset of patients with HP presents circulating present autoantibodies, without features of autoimmune disease (21, 60). In both studies, the presence of autoantibodies was an independent predictor of increased mortality. Patients carrying the haplotype DRB1^*^03:01-DQB1^*^02:01, which is part of the 8.1 ancestral haplotype, and a major genetic determinant of autoimmune diseases showed a significant higher risk to develop autoantibodies (21).

Several studies have reported acute exacerbation (AE) in patients with fibrotic HP, following the same definition used in IPF, which results in poor prognosis (61). Recently it was reported as risk factors lower DLco, the presence of UIP-like pattern on HRCT at diagnosis, and cumulative incidence rates of AE showed high in-hospital mortality rate (62).

Finally, some demographic characteristics, such as aging and smoking may contribute to disease progression (3, 19).

## Therapeutic Approaches

There is no consensus or guidelines for HP management, and most of the evidence arises from retrospective cohort studies or case reports. The therapeutic approach consists mainly of antigen avoidance and pharmacological treatment with corticosteroids/immunosuppressive drugs and, more recently, antifibrotic therapy depending on HP phenotype. In advanced disease with severe clinical and functional deficiency, a lung transplant is indicated.

### Antigen Avoidance

Identification and complete antigen avoidance are the mainstays of treatment and patients should be strongly advised to avoid further exposure (1, 19, 30). However, in 40–50% of HP patients the antigen(s) is not identified.

In non-fibrotic HP antigen-avoindance is associated with improved lung function but in fibrotic forms, the effectiveness remains controversial (63). In a cohort of patients with fibrotic HP, FVC remained stable and median survival was greater in patients who reported antigen avoidance while in another study no difference was found suggesting a self-perpetuating mechanism of the disease in fibrotic forms (63, 64). Despite these observations, it is important to make continuous efforts to identify the antigens' source and strongly recommend avoid exposure.

## Pharmacological Treatment

### Non-fibrotic HP

Corticosteroids are often used but the evidence supporting this approach is very limited and comes from studies in farmers lung disease, where pulmonary function improved during early follow-up protecting against progression but without beneficial effect on long term prognosis (65, 66). Recently De Sadeleer et al., showed that corticosteroid initiation in progressive patients resulted in a reversal with an improvement of lung function (63). An empiric treatment scheme may consist of prednisone (or equivalents) of 0.5 mg/kg/d for 1–2 weeks followed by a gradual tapering until maintenance of 10 mg/d (67). To ameliorate adverse events related to the prolonged corticosteroid use, sparing agents, mycophenolate (MMF) and azathioprine (AZA), might be a treatment option for patients showing progression and/or frequent relapses or in whom antigen avoidance is not possible.

### Fibrotic HP

For many reasons, pharmacological treatment in fibrotic HP is challenging. Despite the lack of evidence, corticosteroids alone or associated with AZA or MMF are the most common immunosuppressants used for treating fibrotic HP, with fewer adverse events with combination therapy. In a retrospective study, a modest but significant improvement in DL_CO_ without changes in FVC was observed after 1-year treatment of MMF or AZA (68). The presence of BAL lymphocytosis seems to be associated with a favorable response to corticosteroids alone or in combination, especially with AZA, but only during the first 6 to 12 months of treatment, with FVC decline after this period (63, 69). HRCT honeycombing, low BAL lymphocytosis, and the presence of short telomeres could be factors associated with no response to immunosuppressive therapy (63, 70). Moreover, treatment with corticosteroids alone or in combination with AZA/MMF was associated with increased mortality risk after adjustment in two cohorts of patients with fibrotic HP. This finding is similar to those reported in IPF, probably reflecting a final common pathway in the pathophysiologic processes of advanced fibrosis that underlies these two diseases (25, 71, 72).

There is emerging evidence that Rituximab, an anti CD20 monoclonal antibody, seems to be well-tolerated and may lead to stabilization or improvement of lung function in some patients with fibrotic HP, particularly those without UIP or NSIP pattern (73, 74). Finally, leflunomide, a prototype member of dihydroorotate dehydrogenase (DHODH) enzyme inhibitors, could be an effective sparing immunomodulatory drug with a significant pulmonary function improvement in fibrotic HP, with a most pronounced effect in patients without >20% extent of fibrosis on HRCT (75).

Antifibrotics, pirfenidone and nintedanib, recently became a plausible option for patients who experience disease progression despite antigen avoidance and immunosuppressive treatment. The efficacy and safety of pirfenidone were evaluated in the RELIEF study that was prematurely terminated due to slow recruitment (76). Despite this, 45% of the patients included in the study had fibrotic HP and the addition of pirfenidone to ongoing medication showed slower disease progression as measured by loss of FVC. This data is similar to another real-life study where pirfenidone reduced the decline of vital capacity in a cohort of patients with fibrotic HP (77). By contrast, in a small cohort of patients with fibrotic, advanced HP, we found that adding pirfenidone to the immunosuppressive drugs, showed no effect on FVC compared with the patients using only immunosuppressive therapy, but displayed a tendency to DLCO improvement and a significant improvement in the quality of life evaluated through the total score on the Saint George's Respiratory Questionnaire (78).

The INBUILD trial demonstrated that in patients with progressive fibrosing interstitial disease the annual rate decline in the FVC was significantly lower among patients who received nintedanib than those who received placebo (79). In the fibrotic HP subgroup (26% of the overall population) there was no statistical difference in the rate of FVC decline between nintedanib and placebo, likely because the study was not designed to provide evidence in specific subgroups (80). Finally, in patients with progressive and severe fibrotic HP, it should be considered for a lung transplant (81).

In summary, the management of HP patients should include, antigen avoidance in both HP phenotypes. For patients with non-fibrotic HP who don't have a full recovery after antigen removal, it is suggested corticosteroid treatment with a gradual tapering to achieve a low dose with or without AZA or MMF for patients with frequent relapses or when antigen avoidance it's not possible. In light of the evidence, in fibrotic HP and preferable after careful evaluation with multidisciplinary team discussion using HRCT, BAL, and histopathology findings to identify those with mixed inflammatory plus fibrotic or purely fibrotic disease, immunosuppressive therapy, and antifibrotic treatment should be considered and in advance disease, patients should be included for a lung transplant.

## Palliative Care

As in many interstitial lung disease that present progressive pulmonary fibrosis phenotype and end-stage disease, the decrease in quality of life of patients with HP represents an additional problem. Quality of life is not only affected by the disease but also by the presence of adverse events associated with the treatment, inability to continue with work or recreational activities and the economic impact for the family. Palliative care should be discussed and initiated early in the disease course, and should be focused not only according patient's needs and preferences but also include caregivers which should be supported throughout the disease trajectory (82).

## Conclusions

The diagnosis and treatment of HP remain complex and challenging because the absence of a single diagnostic gold standard and lack of prospective clinical trials.

For a long time, HP was characterized by duration of symptoms at the time of diagnosis, as acute, subacute, or chronic which was not reliably associated with the prognosis. Consequently, a recently published guideline has proposed that patients should be classified as having fibrotic or non-fibrotic HP, according to the radiological or histopathological findings. These two phenotypes are clearly identifiable and likely show a better association with outcome. The pathogenic mechanisms have not been fully elucidated, and diagnostic and prognostic biomarkers are lacking. The prognosis of fibrotic HP is poor as in other fibrotic lung disorders, and questions remain unanswered about the optimal therapeutic strategy mainly for fibrotic HP for which large-scale clinical trials are necessary.

## Author Contributions

MS: conceptualization and writing the first draft. IB-R, MA, and ER-A: review and writing. All authors contributed to the article and approved the submitted version.

## Conflict of Interest

The authors declare that the research was conducted in the absence of any commercial or financial relationships that could be construed as a potential conflict of interest.

## Publisher's Note

All claims expressed in this article are solely those of the authors and do not necessarily represent those of their affiliated organizations, or those of the publisher, the editors and the reviewers. Any product that may be evaluated in this article, or claim that may be made by its manufacturer, is not guaranteed or endorsed by the publisher.
